# Acute appendicitis in patients suffering from Immunocompromising hematologic malignancies or chemotherapy-related leukopenia: a cohort study

**DOI:** 10.1186/s12885-025-14493-2

**Published:** 2025-07-01

**Authors:** Alexandra C. Klein, Hannah Rasel, Oliver Kriege, Fabian Bartsch, Hauke Lang, Ann-Kathrin Lederer

**Affiliations:** 1 Department of General, Visceral and Transplant Surgery, University Medical Centre of the Johannes Gutenberg University, Langenbeckstraße 1, 55131 Mainz, Germany; 2 Department of Medicine III, University Medical Centre of the Johannes Gutenberg University, 55131 Mainz, Germany; 3 Department of General and Visceral Surgery, Hospital “Barmherzige Brüder” Trier, 54292 Trier, Germany

**Keywords:** Hematologic diseases, Postoperative complication, Surgery, Leukocyte disorders, Neutropenia, Mortality, Morbidity

## Abstract

**Background:**

Acute appendicitis is a common surgical emergency, but its diagnosis can be challenging in patients with hematologic malignancies or chemotherapy-induced leukopenia due to an impaired inflammatory response. The clinical presentation of acute appendicitis in these patients is often atypical, leading to delayed or misdiagnosis. This study aims to evaluate the outcomes of appendectomy in patients with hematologic malignancies or chemotherapy-induced leukopenia and assess the applicability of clinical scoring systems for the diagnosis of an acute appendicitis.

**Methods:**

We conducted a retrospective analysis of patients with hematologic malignancies or chemotherapy-related leukopenia who underwent appendectomy for suspected acute appendicitis between 2007 and 2023. Clinical presentation, laboratory findings, and imaging results were reviewed. The accuracy and relevance of clinical scoring systems for diagnosing acute appendicitis in these patients were also evaluated.

**Results:**

Our study included 12 patients with hematologic malignancies or chemotherapy-induced leukopenia who underwent appendectomy. Atypical clinical presentations were common, with a lower frequency of fever, elevated leukocytes, and other typical inflammatory markers. Only one patient developed postoperative complications (acute kidney failure), and none of the patients died due to appendicitis. Clinical scoring systems demonstrated limited applicability in this patient population, often underestimating the likelihood of appendicitis.

**Conclusion:**

Diagnosing acute appendicitis in patients with hematologic malignancies or chemotherapy-induced leukopenia poses significant challenges, as standard scoring systems prove unreliable. The presence of abdominal pain coupled with elevated C-reactive protein (CRP) levels should prompt a multidisciplinary evaluation and timely imaging. Surgical therapies, particularly laparoscopic approaches, appear safe and feasible in these patients. Ongoing research is essential to refine surgical strategies for the growing population of immunocompromised individuals.

## Background

Acute appendicitis is one of the most common emergencies in adult visceral surgery, most frequently affecting individuals in the second to fourth life decade [1]. The annual incidence is almost 100 cases per 100 000 adults [2, 3]. Typically, patients present with a progressive right lower quadrant pain, which is usually accompanied by a deterioration in general condition, anorexia, fever, nausea and vomiting [1, 4]. Every second patient reports about a migration of pain from the upper to the lower abdomen [4, 5]. Clinical examination may reveal rebound tenderness and muscular defence, primarily in the lower right quadrant [6]. Ultrasound examination can confirm the suspected diagnosis with a positive predictive value of more than 90% [7, 8]. In unclear cases, a computed tomography (CT) scan is usually performed, as CT has a high sensitivity and specificity for the diagnosis of acute appendicitis [4, 9–11]. Laboratory tests show elevated inflammatory parameters such as leukocytosis and increased C-reactive protein (CRP), often accompanied by a left shift in the white blood cell count [4]. However, the diagnosis of acute appendicitis can be challenging in certain populations, including the elderly and patients with underlying comorbidities [12, 13]. Misdiagnosis or delayed diagnosis may lead to complications such as perforation or sepsis, while overdiagnosis may result in unnecessary surgery [14–16]. The diagnosis of acute appendicitis is therefore facilitated by various scoring systems such as the Alvarado-Score or the Appendicitis Inflammatory Response (AIR). These systems rely heavily on the presence of inflammatory signs and laboratory markers, which may be absent or blunted in immunocompromised patients [17, 18]. Previous studies, including a small pediatric case series, have highlighted the difficulty of diagnosing appendicitis in immunocompromised patients [19]. However, data on adult patients remain scarce. The aim of this study was to evaluate the clinical presentation, diagnostic workup, and outcomes of adult patients with hematologic malignancies or chemotherapy-induced leukopenia who underwent appendectomy for suspected acute appendicitis. We specifically sought to assess the applicability of clinical scoring systems and laboratory parameters in this patient population.

## Methods

We performed a monocentric retrospective cohort study at the Department of General, Visceral and Transplant Surgery of the University Medical Centre Mainz. Data were obtained from the electronic database of all patients who underwent surgery between 2007 and 2023. The primary aim of the study was the outcome of patients with disorders of the immune systems including hematologic malignancies who underwent appendectomy for acute appendicitis. We initially hypothesized that the outcome of these patients would be worse compared to an otherwise healthy control group. As a secondary aim of the study, we aimed to evaluate the applicability of clinical scoring systems for acute appendicitis in patients with hematologic malignancies or leukopenia. Eligible for inclusion were patients diagnosed with the International Statistical Classification of Diseases and Related Health Problems (ICD, 10th Revision) code for different immune-comprising hematologic malignancies or for leukopenia (see Table 1) [20] who underwent appendectomy. Only patients with a diagnosis of acute appendicitis leading to appendectomy were eligible for inclusion. Patients who underwent another resection with simultaneous appendectomy were excluded. Patients who were diagnosed with other abdominal diseases with a concomitant appendicitis were also excluded. The probability of an appendicitis diagnosis was estimated using different scoring systems (see Table 2.). If documentation for a score-related parameter was missing, no points were counted for this item, as it was assumed that the corresponding symptom was not present. Complications were rated according to the Clavien-Dindo classification [21, 22].

Study size depended on feasibility and was not calculated as acute appendicitis in patients suffering from hematologic malignancies or leukopenia is a rare disease. Due to the retrospective study character, no ethical approval was required. Before the start of the study, we planned to create a control group to compare the outcome of patients with hematologic malignancies or leukopenia with otherwise healthy patients, but after a first evaluation of results, we waived comparison due to the mostly complication-free course of the patients. To estimate the incidence of acute appendicitis in patients with immunocompetent hematologic malignancies, we estimated the total number of patients treated in our clinic during the period with one of the diseases listed in Table 1 using data from the cancer registry. Numerical data are presented as mean values and standard deviation unless otherwise stated.

**Table 1:** Diagnoses by the code of the international statistical classification of diseases and related health problems (ICD, 10th Revision) [20], which were used for Raw data obtaining

ICD	Disease
D46	Myelodysplastic syndromes
D47	Other neoplasms of uncertain or unknown behaviour of lymphoid, hematopoietic and related tissue
D70	Agranulocytosis
D72	Other disorders of white blood cells
C81	Hodgkin lymphoma
C82	Follicular lymphoma
C83	Non-follicular lymphoma
C84	Mature T/NK-cell lymphomas
C85	Other and unspecified types of non-Hodgkin lymphoma
C86	Other specified types of T/NK-cell lymphoma
C88	Malignant immunoproliferative diseases
C90	Multiple myeloma and malignant plasma cell neoplasms
C91	Lymphoid leukaemia
C92	Myeloid leukaemia
C93	Monocytic leukaemia
C94	Other leukaemia of specified cell type
C95	Leukaemia of unspecified cell type
C96	Other and unspecified malignant neoplasms of lymphoid, hematopoietic and related tissue

**Table 2:** Clinical scoring systems for diagnosis of acute appendicitis

Alvarado score for acute appendicitis [17]:
The minimum score of Alvarado score is 0 points, the maximum score is 10 points
Acute appendicitis is likely at AIR score of at least 6 points.
- right lower quadrant tenderness (no vs. yes (= 2 points))
- elevated temperature (>37.3°C) (no vs. yes (= 1 point))
- rebound tenderness (no vs. yes (= 1 point))
- migration of pain to the right lower quadrant (no vs. yes (= 1 point))
- loss of appetite (no vs. yes (= 1 point))
- nausea or vomiting (no vs. yes (= 1 point))
- leukocytosis (>10,000) (no vs. yes (= 2 points))
- leukocyte left shift (>75% neutrophils) (no vs. yes (= 1 point))
Appendicitis Inflammatory Response (AIR) score [18]:
The minimum score of AIR is 0 points, the maximum score is 12 points.
Acute appendicitis is likely at AIR score of at least 8 points.
- vomiting (no vs. yes (= 1 point))
- right iliac fossa pain (no vs. yes (= 1 point))
- round tenderness (light = 1 point, medium = 2 points, strong = 3 points)
- elevated temperature (38.5°C) (no vs. yes (= 1 point))
- polymorphonuclear leukocytes (70-84% = 1 point, ≥85% = 2 points)
- leukocytosis (>10,000 = 1 point, ≥15 000 = 2 points)
- CRP level (mg/dl) (10-49 = 1 points, ≥ 50 = 2 points)
RIPASA score fur acute appendicitis [23]:
The minimum score of RIPASA is 1.5 points, the maximum score is 17 points.
Acute appendicitis is likely at AIR score of at least 7.5 points.
- sex (female = 0.5 points, male = 1 point)
- age (≤40 years of age = 1 point, >40 years of age = 0.5 points)
- foreign nationality (no vs. yes (= 1 point))
- right iliac fossa pain (no vs. yes (= 0.5 points))
- migration of pain to the right lower quadrant (no vs. yes (= 0.5 points))
- anorexia (no vs. yes (= 1 point))
- nausea or vomiting (no vs. yes (= 1 point))
- duration of symptoms (≤ 48 hours = 1 point, >48 hours = 0.5 points)
- right iliac fossa rebound tenderness (no vs. yes (= 1 point))
- guarding (no vs. yes (= 2 point))
- rebound tenderness (no vs. yes (= 1 point))
- Rovsing’s sign (no vs. yes (= 2 point))
- temperature between 37 and 39°C (no vs. yes (= 2 points))
- elevated white blood cell count (no vs. yes (= 1 point))
- negative urine analysis (no vs. yes (= 1 point))

## Results

**Fig. 1:**
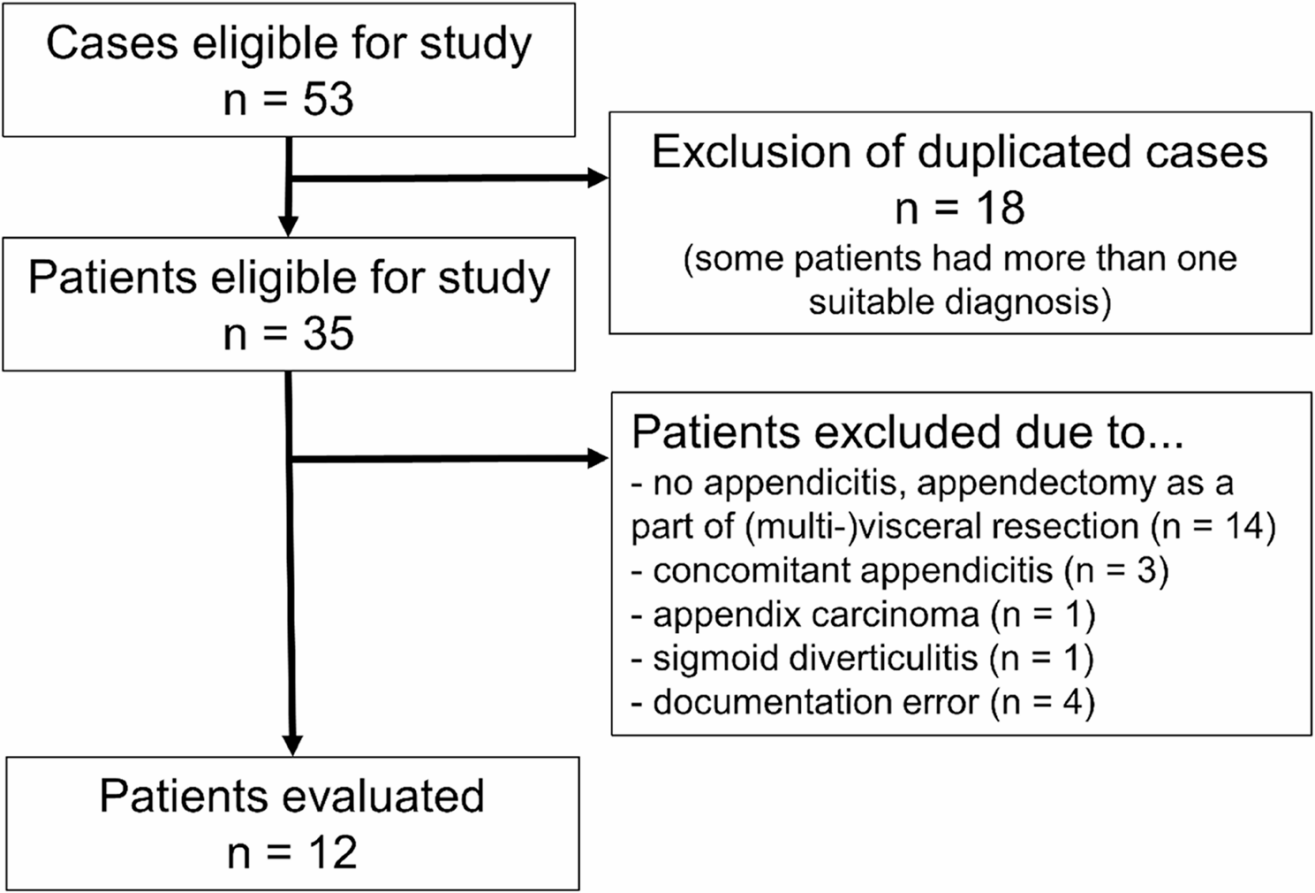
Overview of selection process

Overall, 12 patients were eligible for study inclusion. An overview of the selection process is shown in Fig. 1.

Demographic patient data are shown in Table 3. Half of the patients were female (*n* = 6, 50%). Patients were on average 44 ± 17.5 years old (range 18–68). All of the patients had a malignant primary disease, mostly hematologic malignancies. Only three patients (25%) had a solid tumour and developed leukopenia during chemotherapy. Most of the patients (*n* = 10, 83%) had a German origin. Hospital stay lasted on average 40.4 ± 43.4 days for further treatment of the underlying disease. More than half of the patients (*n* = 8, 67%) were not treated on a surgical ward postoperatively.

**Table 3:** Overview of patients’ demographic data

	**Sex**	**Age**	**Diagnosis**	**Year of treatment**	**Origin**	**First presentation**	**Duration of hospital stay** (days)	**Stay on surgical ward **(days)	Time to surgery*(hours)
1	m	58	AML	2007	Germany	internal medicine	154	0	120
2	f	58	AML	2008	Germany	internal medicine	35	0	21
3	f	48	mamma carcinoma	2008	Germany	surgical ER	8	8	2
4	f	68	endometrial cancer	2008	Germany	internal medicine	10	6	0
5	m	53	AML	2013	Germany	internal medicine	41	0	4
6	m	40	germ cell tumour	2011	Columbia	internal medicine	23	0	13
7	f	48	ALL	2013	Germany	internal medicine	61	0	30
8	f	18	AML	2014	Romania	internal medicine	31	0	18
9	m	19	B-cell lymphoma	2018	Germany	internal medicine	5	5	1
10	m	62	AML	2021	Germany	internal medicine	21	0	4
11	m	33	B-cell lymphoma	2010	Germany	surgical ER	8	8	0
12	f	21	AML	2023	Germany	Internal medicine	88	0	4

*ER* emergency room, *m* male, *f* female, *AML* acute myeloid leukaemia, *ALL* acute lymphatic leukaemia, *n.s* not specified

*time between first symptoms and surgery

The time to surgery (first symptoms to surgery) was on average 18.1 ± 33.5 h. Diagnostic data are shown in Table 3. In 9 patients (75%) diagnosis was made by computed tomography. Almost all of the patients (*n* = 11, 92%) had abdominal pain. Only half of the patients (*n* = 7, 58%) stated pain in the lower right quadrant. None of the scorings were able to predict acute appendicitis as all of the summed values were lower than the cut-off values making acute appendicitis unlikely. Only one patient (8%) showed an evaluable leukocytosis, further eight patients (67%) had leukopenia (mostly grade 4) and one patient (8%) suffered from massive leukocytosis due to his primary hematologic disease. CRP was elevated in all patients. There was a lack of documentation, especially for the occurrence of Rovsing’s sign (100% missing), loss of appetite (92% missing), for leukocyte left shift (92% missing) and fever (58% missing).

**Table 4:** Overview of diagnostic measurements

	**Diagnosis by**	**Pain in the lower right abdomen**	**Alvarado** **Score**	**AIR Score**	**RIPASA Score**	**CRP **(mg/l)	**Leukocytes **(thousand cells/µl)	**Leukopenia**	**Grade of leukopenia** ^**+**^	**Reason for leukopenia**
1	CT scan	yes	2	3	2	190	0.7	yes	4	chemotherapy
2	CT scan	no	0	2	1	159	0.6	yes	4	chemotherapy
3	clinical	yes	3	6	1.5	110	2.3	yes	2	chemotherapy
4	ultrasound	yes	2	3	1.5	82	0.9	yes	4	chemotherapy
5	CT scan	no	1	4	1.5	171	47.3	no	no	no
6	CT scan	yes	2	3	3	134	0.26	yes	4	chemotherapy
7	CT scan	no	2	0	2	9.7	11.1	no	no	no
8	CT scan	yes	3	3	3.5	248	0.24	yes	4	chemotherapy
9	CT scan	yes	4	5	3.5	81	1.33	yes	3	chemotherapy
10	CT scan	no	1	3	2	301	0.52	yes	4	MDS
11	clinical	n. s.	0	2	2	214	n. s.	n. s.	n. s.	n. s.
12	CT scan	yes	2	3	5	137	0.2	yes	4	chemotherapy

Alvarado score [17], AIR score [18], RIPASA Score [23], ^+^Grade of leukopenia according to Palmblad et al. and the National Cancer Institute [24, 25]; *CT* computed tomography

Table 4 shows an overview of surgery-related data. Six patients (50%) had laparoscopic surgery. Complicated appendicitis (defined as intraoperative finding of perforation or abscess) was found in three patients (25%). None of the patients developed clinically evident abscess or wound infection postoperatively. Only one patient (8%) had a postoperative complication, an acute kidney failure. None of the patients died due to acute appendicitis. The pathologist confirmed the diagnosis of appendicitis in all patients, with three patients showing primarily chronic changes. All of the patients with chronic as opposed to acute changes had leukopenia. In one patient with acute myeloid leukaemia, infiltration of the appendix by blasts was described in addition to acute appendicitis.

**Table 5:** Overview of patients’ surgical-related data

	**Surgery**	**Approach**	**Abscess**	**Perforation**	**Deceased**	**Deceased due to appendicitis**	**Complication**	**Grade of complication**	**Type of complication**
1	appendectomy	open	no	no	yes*	no	no		
2	appendectomy	open	no	no	no	no	no		
3	appendectomy	open	no	no	no	no	no		
4	appendectomy	open	no	yes	yes*	no	no		
5	appendectomy	open	no	no	yes*	no	yes	IV	septic kidney failure
6	appendectomy	laparoscopic	no	no	no	no	no		
7	appendectomy	laparoscopic	yes	no	no	no	no		
8	appendectomy	laparoscopic	no	no	no	no	no		
9	appendectomy	laparoscopic	no	no	no	no	no		
10	appendectomy	laparoscopic	no	no	no	no	no		
11	appendectomy	open	yes	yes	no	no	no		
12	appendectomy	laparoscopic	no	no	yes*	no	no		

*Later death due to pulmonary sepsis (patient 5) and primary disease progression (patient 1, 6 and 13)

In our study, nine patients (75%) suffered from hematologic malignancies. A total of 7108 patients with hematologic malignancies were treated at our clinic during the study period. The incidence of acute appendicitis in the whole cohort of patients with hematologic malignancies was 0.13%.

## Discussion

This is one of the first cohort studies to investigate the clinical presentation and the outcome of acute appendicitis in patients with immunocompromising hematologic malignancies or with a chemotherapy-induced leukopenia. The results suggest that appendectomy might be a safe approach in acute appendicitis in this vulnerable cohort as almost all of the patients had a complication free postoperative course. The only postoperative complication in our cohort was septic renal failure. This may have resulted from a four-day delay before surgery. The results underscore the difficulty of diagnosis, as the commonly recognized clinical signs of appendicitis were absent in most of our patients. Likewise, the scoring systems used for appendicitis could not confirm the diagnosis. The results are limited due to the small sample-size and the retrospective nature of the study, which always carries the risk of documentation errors. As some of the diagnostic features required by the appendicitis scoring systems were not documented, we had to assume that these signs were not present in the patients. This assumption could, of course, be wrong, since the lack of documentation could also mean that these points were not questioned or that the documentation was forgotten. The rating systems for appendicitis are not new, as Alvarado, for example, introduced his system in the mid-1980s [17]. However, the World Society of Emergency Surgery (WSES) recommends the use of scoring systems, but only for ruling out acute appendicitis and identifying intermediate-risk patients. The WSES guideline emphasizes that the Alvarado score may lack specificity and be unreliable in distinguishing between complicated and uncomplicated appendicitis, especially in sensitive cohorts such as the elderly and patients with HIV [26]. Due to impaired inflammatory responses in patients with hematologic malignancies or chemotherapy-induced leukopenia, traditional appendicitis scoring systems may fail, as also highlighted by WSES guidelines [27–30]. The mechanism of inflammatory response is a complex and sensitive equilibrium, which can be easily destabilized by several diseases or disease-related drugs [31]. Most of the other signs of acute appendicitis such as fever and deterioration of general condition are also caused by the inflammatory response of the body. It is therefore questionable whether typical signs of acute appendicitis are to be expected in patients with hematologic malignancies or with chemotherapy-induced leukopenia. A study by Hobson et al. evaluated seven cases of acute appendicitis in children with hematologic malignancies emphasizing the challenge of diagnosis as five of seven children were clinically misdiagnosed due to an atypical presentation [19]. In 1993, Kim et al. reported four cases of acute appendicitis in leukaemia patients [32]. Unlike our patients, all of the patients in this case series reported pain in the right lower quadrant. Another case report by Forghieri et al. discusses a patient with multiple myeloma and a patient with acute myeloid leukaemia [33]. The first patient presented with an atypical clinical picture of diffuse abdominal pain, nausea and vomiting without fever, which later led to a computed tomography scan with the diagnosis of an acute appendicitis. The second patient reported lower abdominal pain and subfebrile temperatures. In both patients, the appendectomy was without complications. In a case report by Kojima et al., two patients with acute appendicitis were evaluated [34]: one with an immediate appendectomy with an uneventful course and one with antibiotic treatment. In the second patient, treating physicians assumed that the bone marrow function of the patients would recover rapidly after discontinuing chemotherapy, which would then allow a planned and safe appendectomy. Unfortunately, the patient did not recover in time and developed a liver abscess 23 days after diagnosis of appendicitis, which led to a laparoscopic exploration and appendectomy. Due to the advanced appendicitis, a partial resection of the cecum was necessary. The postoperative course was uneventful. Chirletti et al. reported on patients with acute abdominal pain suffering from hematologic disease [35]. Five of the patients with acute myeloid leukaemia (AML) had acute appendicitis and underwent appendectomy. Abdominal tenderness was present in all patients, but localized in three patients, while diffuse pain was present in two other patients. Three of the patients experienced complications, which were not further defined. In 2023, Lee et al. reported on 89 patients with various hematologic disorders with the diagnosis of acute appendicitis [36]. Out of these, 75 received appendectomy. The authors found no association between either hematologic disease or preoperative laboratory findings and surgical complications. Lee et al. also examined 14 patients who did not undergo surgery despite appendicitis and who died more often than the operated patients (14 vs. 3%). Typical clinical and laboratory signs, including right lower abdominal pain, appear to be unreliable for diagnosing appendicitis in these patients. Although most patients reported abdominal pain, classic symptoms were often absent, leading to poor scoring system performance. Abdominal discomfort and pain due to side effects are common symptoms in patients undergoing chemotherapy, which makes the diagnosis of acute appendicitis even more difficult. The WSES indicates that CRP could be of fundamental importance in immunocompromised patients to diagnose abdominal infections [30]. In comparison with other inflammatory markers, CRP showed a greater reliability as it was elevated in all of our patients. In general, CRP is a non-specific acute-phase protein, which is often elevated in patients with cancer, sometimes even to levels similar to those in the patients in our study [37, 38]. However, various types of activated cells, e.g. endothelial cells, can produce cytokines such as interleukin-1 or interleukin-6, which ensure that there is an increased production of acute-phase proteins in inflammation [39]. CRP is thus not only influenced by the cellular immune system, but also by other cells in the body, which could make it a helpful tool for diagnosing acute appendicitis in immunocompromised patients. Of course, CRP is not an explicit indication of acute appendicitis, but the combination of increased CRP and abdominal pain in patients with hematologic malignancies or leukopenia should make one think of the possibility of an acute abdominal infection or even appendicitis. In these patients, the combination of abdominal pain and elevated CRP should raise suspicion and prompt an examination using ultrasound or CT scan. In most of the above-mentioned studies, the diagnosis was made using ultrasound or CT scan, which is consistent with our results [33–35].

The estimated incidence of acute appendicitis in our study showed that acute appendicitis could occur with a similar rate in patients with hematologic malignancies or chemotherapy-related leukopenia as in the general population. However, it should be borne in mind that there may be patients who were not diagnosed with acute appendicitis due to non-specific clinical symptoms. It is unclear whether there were patients who were not referred to us and who were treated with antibiotics by the colleagues in internal medicine for an unclear abdominal infection. In recent years, there has been much debate about whether appendicitis can be treated conservatively with antibiotics instead of surgery. A large randomized trial compared both approaches in the general patient population and found conservative treatment non-inferior to surgery as measured by the European Quality of Life–5 Dimensions (EQ-5D) questionnaire at 30 days [40]. However, conservatively treated patients had more complications and 29% underwent appendectomy within 90 days. Within three years after randomization, 49% of the conservatively treated patients had undergone appendectomy [41]. International guidelines on acute abdomen in immunocompromised patients recommend appendectomy over conservative treatment in transplanted patients [30]. Chirletti et al. emphasized that an optimal outcome in patients with hematologic diseases requires rapid initiation of therapy and, if necessary, replacement of blood components. A patient’s critical condition should not be a reason to forgo surgical therapy [35]. In our cohort, it lasted approximately one day until surgery. There is debate about how much time should pass between the diagnosis of appendicitis and appendectomy [42, 43]. However, it is difficult to transfer the scientific basis of the debate to our patients because it is unclear how a time delay applies to immunocompromised patients. Moreover, in our study, the patients did not wait for their operation, but for the diagnosis. It is therefore unclear whether they have already received antibiotic treatment, for example. Given the significant uncertainty in the diagnosis of appendicitis in patients with hematologic malignancies or leukopenia, a high degree of alertness should be maintained in immunocompromised patients with abdominal symptoms. As a clinical consequence, a multidisciplinary discussion of the patient is recommended to utilize the expertise of internal medicine, surgery and radiology to reach diagnosis. As the results of surgery in our study were favourable with a low complication rate and results of other studies indicate similar trends, surgery should be preferred over conservative treatment in patients with appendicitis suffering from hematologic disease or chemotherapy-related neutropenia. This is expected to ensure rapid infection control and avoid complications such as rupture of the appendix, which could be catastrophic for immunocompromised patients.

## Conclusion

The diagnosis of acute appendicitis in patients with immunocompromising hematologic malignancy or chemotherapy-induced leukopenia remains challenging. Commonly used scoring systems for acute appendicitis are unreliable in diagnosing appendicitis in these patients. The combination of abdominal pain and elevated CRP should lead to a multidisciplinary discussion and, if in doubt, to immediate imaging. In case of acute appendicitis, surgery seems feasible and safe, even when performed laparoscopically. Future work should continue to address surgical therapies in immunocompromised patients, since a relevant proportion of the general population belongs to this group due to diseases or side effects of therapies.

## Data Availability

Data is provided within the manuscript.

## Abbreviations

AIR: Appendicitis Inflammatory Response
ALL: Acute lymphatic leukaemia
AML: Acute myeloid leukaemia
CRP: C-reactive protein
CT: Computed tomography
ER: Emergency room
EQ-5D: European Quality of Life–5 Dimensions
f: Female
HIV: Human immunodeficiency virus
ICD: International Statistical Classification of Diseases and Related Health Problems
m: Male
n.s.: not specified
WSES: World Society of Emergency Surgery
